# Dermoscopy of oral and genital mucosal lesions: A descriptive cross-sectional study protocol

**DOI:** 10.1371/journal.pone.0289562

**Published:** 2023-08-15

**Authors:** Ajay Dodeja, Sushil Pande, Bhushan Madke

**Affiliations:** 1 Department of Dermatology, Venerology, and Leprology, NKP Salve Institute of Medical Sciences & Research Centre and Lata Mangeshkar Hospital, Nagpur, Maharashtra, India; 2 Department of Dermatology, Venerology, and Leprology, Jawaharlal Nehru Medical College, Datta Meghe Institute of Higher Education and Research, Wardha, Maharashtra, India; Affiliated Hospital of Nanjing University of Chinese Medicine, CHINA

## Abstract

**Introduction:**

Dermoscopy is a safe, rapid, and non-invasive tool that aids in the clinical examination of pigmented and non-pigmented lesions. The upward trend in the use of dermoscopy can be attributed to the availability of compact hand-held and sophisticated dermoscopes, that are small enough to be carried around in a pocket. The extent of dermoscopy is not only limited to the evaluation of cutaneous lesions but also involves its use in the assessment of mucosal lesions along with lesions of hair and nails.

**Methods:**

In a descriptive cross-sectional study, subjects (n = 100) with oral or genital mucosal lesions will be enrolled. Following a thorough clinical examination, a dermoscopy of the lesion will be performed with Dermlite DL4© Dermoscope, having a magnification of 10x. Images obtained would be stored and evaluated for observing specific morphologic patterns on dermoscopy which would be utilized to describe those patterns and arrive at a specific diagnosis. Descriptive statistics will include mean and standard deviation to summarise quantitative variation. Dermoscopic features of oral and genital mucosal lesions will be estimated in percentage.

**Purpose of study:**

Mucosal lesions several times mimic each other morphologically. Performing a biopsy is not always feasible for oral and genital lesions because they may be difficult to reach and tend to bleed more profusely compared to the skin surface due to its rich vascular nature. Dermoscopy is a non-invasive tool that helps in the diagnosis that is used mostly for the evaluation of non-mucosal lesions. For the same reason, there is no or minimal information in the published literature with regard to dermoscopic patterns of mucosal lesions. The current study intends to describe dermoscopic patterns in oral and genital mucosal diseases so that this important information would assist the diagnosis in a non-invasive manner thereby reducing the need for invasive investigations like mucosal biopsy.

**Expected clinical outcomes:**

To summarize, this research is intended to add to the scarce literature on dermoscopic findings of oral and genital mucosal lesions. The study findings would establish the diagnosis and eliminate the need for unwarranted invasive biopsies of mucosal lesions and, if need be, help in the selection of the biopsy site.

## Introduction

Within the field of dermatology, a non-invasive method called dermoscopy or, dermatoscopy, even referred to as epiluminescence microscopy and skin surface microscopy, finds its application to assess skin, hair, nail, and mucosal lesions with the help of a handheld instrument known as dermoscope. Mucoscopy remains to be an inadequately explored facet of dermatology to evaluate mucosal surfaces employing a dermoscope [1, 2]. Dermoscope can serve as a non-invasive modality when manoeuvred by a proficient dermatologist to interpret several clues including the vascular pattern, the colour, and follicular changes in several inflammatory, infectious and neoplastic skin conditions which eventually facilitates the diagnosis [3, 4].

Cases with mucosal lesions account for a major portion of standard dermatological practice, and histopathological confirmation is neither possible nor desirable in all cases. Not only is mucosal biopsy more challenging, but also possesses higher risk of bleeding. Dermoscopy possesses the capacity to not only bring down the need for performing biopsies, but also helps in selecting site of biopsy in case inevitable.

There was a sense of caution among clinicians regarding the application of dermoscopy on mucosal lesions due to the possibility of infection arising from direct contact between the dermoscope and the mucosa. Nevertheless, these concerns have been overcome through innovative approaches such as the utilization of cling films and barrier footplates [5, 6]. The primary purpose of this descriptive cross-sectional study was to investigate the dermoscopic features exhibited by oral and genital mucosal lesions upon their initial presentation.

## Materials and methods

The study proposal was approved by Institutional Ethics Committee (Re-Registration No.: ECR/88/Inst/MH/2013/RR/19) on 14^th^ September 2022 with reference number: 104/2022.

All patients irrespective of age and gender attending Dermatology, Venereology & Leprosy outpatient & in patient department of a tertiary care hospital will be included in the study over the period of two years.

Sample size is determined considering proportion of infectious genital diseases as the main outcome measure. Following assumptions are made from similar study conducted by Maatouk I et al. (2021). [7]

Expected proportion (p) = 34.75%
Absolute precision (d) = 10%
Desired confidence level (1-α) % = 95%
Therefore, Z = 1.96

$$n=\frac{Z^{2}1-\frac{\alpha }{2}p(1-p)}{d^{2}}$$

By substituting the values in above formula to obtain the sample size, n = 87; Therefore 100 patients with mucosal lesions will be recruited in the proposed study.

100 consecutive study subjects with mucosal lesions who fulfil the inclusion and exclusion criteria and are seeking treatment and care at the study site will be selected by convenient sampling method. Identifying information of the study subjects will be blinded by the authors before the data is shared to any third party.

The study aims to include all the patients who provide informed consent, regardless of age or gender, and with oral or genital mucosal lesions, either with or without concurrent cutaneous involvement, and who are willing to undergo dermoscopic evaluation.

Patients presenting with severely painful lesions, lesions at inaccessible site on oral and genital mucosa, pregnant women and female patients in bleeding phase of menstrual cycle, non-consenting or uncooperative patients will be excluded from the study.

Dermoscopy of oral and genital mucosal lesions will be performed using a handheld dermoscope—Dermlite DL4; 4^th^ generation, with a magnification of 10x. Images will be recorded directly by attaching the dermoscope to iPhone XR with the help of MagnetiConnect^®^Clamp. Snap on disposable IceCap^®^ infection control caps will be used for contact dermoscopy to avoid cross contamination. Both polarised and non-polarised modes will be used to capture various dermoscopic features of mucosal disorders.

Written Informed Consent will be obtained from the patients, who will be included in our study. We will initially take patient details in the Case Record Form and perform dermoscopy to observe dermoscopic features of the lesion. In all patients, a meticulous evaluation will be carried out, encompassing a detailed medical history and clinical examination, with particular attention given to the examination of the mucosal area, including the lips up to the vermilion border. The recorded observations will be documented utilizing a standardized pre-established form. Wherever needed for confirmation of diagnosis, appropriate laboratory investigations such as Tzanck smear, Giemsa staining, Gram staining, Skin biopsy, Direct immunofluorescence (DIF), etc will be carried out.

Lesion site will be cleaned by 0.9% normal saline & dermoscopy will be performed. Use of povidone-iodine solution for cleaning of lesion site will be avoided as it may cause discolouration of the lesion thereby yielding faulty images. Snap on disposable IceCap^®^ will be used instead of plastic cling film for prevention of cross contamination as plastic cling film may yield reflections and cause hindrance while capturing images.

The principal investigator will keep the Case Record Form in a file. All findings will be entered in Microsoft Excel Sheet. The principal investigator will then review the entered data and backup the file. All images captured using iPhone XR will be periodically backed up on an external hard drive. Data will be evaluated periodically. Data will be kept confidential and utilised for further analysis. Data will be coded and analysed in a statistical software STATA version 10.1, 2011 by STATA CORP, TEXAS, USA.

Descriptive statistics will include mean and standard deviation to summarise quantitative variation and frequency or percentage to summarise qualitative variables. Dermoscopic features in oral and genital mucosal lesions will be estimated as percentage.

### Study timeline

#### Data collection

November 2022—April 2024.

#### Statistical analysis & writing of research project

May 2024 –October 2024.

## Discussion

Dermoscopy is the examination of skin lesions with a dermoscope. At its core, dermoscopy operates on the fundamental principle of illuminating a lesion and closely examining it under high magnification to observe subtle features [8]. It enables the examination of skin lesions without interference from surface reflections on the skin. Dermoscopy has been gaining popularity as a non-invasive procedure for the diagnosis of different pigmentary and inflammatory disorders. The utility of dermoscopy as an auxiliary tool in diagnose various mucosal lesions is being explored but is still underutilised. Oral and genital mucosal lesions are often diagnosed clinically while histopathology examination of skin biopsy is the gold standard for diagnosis. Performing a biopsy is not always feasible for oral and genital lesions because the lesions may be difficult to reach and may also tend to bleed more profusely compared to the skin surface due to its rich vascular nature. Hence, dermoscopy—which is a non-invasive tool, is needed to aid prompt and accurate diagnosis of oral and genital mucosal lesions, thereby allowing more efficient management.

A lot of mucosal lesions appear similar clinically while other methods of confirmation are either invasive or have low sensitivity and specificity. Hence, it is essential to know the specific dermoscopic features of oral and genital mucosal lesions.

Dermoscopy is widely practiced in the western countries mostly for the diagnosis of pigmented lesions particularly melanoma in Caucasian skin through non-invasive means. However, in a country like India where inflammatory cutaneous or mucosal lesions are more prevalent than neoplastic pigmented lesions, use of dermoscopy is more frequent for the diagnosis of such lesions. Although there are many studies for use of dermoscopy in cutaneous or non-mucosal lesions, there is scarcity of the literature regarding dermoscopy in mucosal lesions. Although dermoscopic patterns for cutaneous lesions are well identified and used for their diagnosis in India, diagnosis of mucosal lesions with the help of dermoscopy is challenging in the absence of well described dermoscopic patterns in mucosal diseases. Dermoscopy is an evolving technique which has marked beginning of a new era in dermatology practice. However, due to paucity of existing literature on dermoscopic features of oral and genital mucosal lesions, this study is designed to provide an overview at various dermoscopic features of diseases having oral and genital mucosal lesions.

## Supporting information

S1 Checklist STROBE statement—Checklist of items that should be included in reports of observational studies. (DOCX)
